# A three-long non-coding RNA-expression-based risk score system can better predict both overall and recurrence-free survival in patients with small hepatocellular carcinoma

**DOI:** 10.18632/aging.101497

**Published:** 2018-07-13

**Authors:** Jingxian Gu, Xing Zhang, Runchen Miao, Xiaohua Ma, Xiaohong Xiang, Yunong Fu, Chang Liu, Wenquan Niu, Kai Qu

**Affiliations:** 1 Department of Hepatobiliary Surgery, The First Affiliated Hospital of Xi’an Jiaotong University, Xi’an 710061, Shaanxi, China; 2 Institute of Clinical Medical Sciences, China-Japan Friendship Hospital, Beijing 100029, China

**Keywords:** small hepatocellular carcinoma, long non-coding RNA, risk score, prognosis

## Abstract

Growing evidence indicates that long non-coding RNAs (lncRNAs) may be potential biomarkers and therapeutic targets for many disease conditions, including cancer. In this study, we constructed a risk score system of three lncRNAs (*LOC101927051*, *LINC00667* and *NSUN5P2*) for predicting the prognosis of small hepatocellular carcinoma (sHCC) (maximum tumor diameter ≤5 cm). The prognostic value of this sHCC risk model was confirmed in TCGA HCC samples (TNM stage I and II). Stratified survival analysis revealed that the suitable patient groups of the sHCC lncRNA-signature included HBV-infected and cirrhotic patients with better physical conditions yet lower levels of albumin and higher levels of alpha-fetoprotein preoperatively. Besides, Asian patients with no family history of HCC or history of alcohol consumption can be predicted more precisely. Molecular functional analysis indicated that PYK2 pathway was significantly enriched in the high-risk patients. Pathway enrichment analysis indicated that the two lncRNAs (*LINC00667* and *NSUN5P2*) associated with poor prognosis were closely related to cell cycle. The nomogram based on the lncRNA-signature for RFS prediction in sHCC patients exhibited good performance in recurrence risk stratification. In conclusion, we identified a novel three-lncRNA-expression-based risk model for predicting the prognosis of sHCC.

## Introduction

Hepatocellular carcinoma (HCC) is one of the most fatal malignancies because of its dramatically growing incidence and related mortality worldwide [1]. One of the biggest challenges facing most clinicians is the early diagnosis and early surgical intervention of HCC to reduce the resultant public health burden [2]. The development of screening techniques and surveillance programs can at least in part curb the ongoing epidemics of HCC [3]. Despite great improvement in therapeutic approaches, the overall survival (OS) and recurrence-free survival (RFS) rates of HCC remain very low, mainly because HCC is a highly heterogeneous malignancy [4–8]. So, there is an urgent need to identify reliable prognostic and predictive markers to increase risk prediction ability and provide information for guiding proper treatment strategies at the individual level.

Long non-coding RNAs (lncRNAs) are a batch of newly-discovered RNA transcripts that that are usually more than 200 nucleotides. The vast majority of lncRNAs are lack of protein-coding ability [9]. Recent studies have found that lncRNAs-encoded peptides played a critical role in biological activities [10,11], and further some lncRNAs regulated a wide range of transcriptionally or post-transcriptionally biological processes [12]. Moreover, an increasing number of lncRNAs were identified to be associated with the initiation, progression and metastasis of cancer at many sites, including the liver [13–15]. The rapid development of RNA sequencing techniques can help unfold the exciting potential of using lncRNAs as potential biomarkers to facilitate the detection, treatment and prognosis of cancer [16,17]. Currently, only a few lncRNAs such as *HOTAIR* and *HULC* have been well characterized in hepatocarcinogenesis [18,19], and emerging studies proposed that lncRNAs might be potentially reliable predictors for HCC clinical outcomes [20–23]. To yield more information, we in this present study aimed to construct a prognostic risk score system based on lncRNAs expression data to predict the prognosis of small HCC (sHCC) through a comprehensive analysis of microarray data. The sHCC is a special type of HCC with the maximum tumor diameter ≤5 cm defined in this study and favorable long-term outcomes, and so early detection of sHCC has very important clinic value.

## RESULTS

### Construction of the prognostic risk score system of sHCC

**Figure 1:**
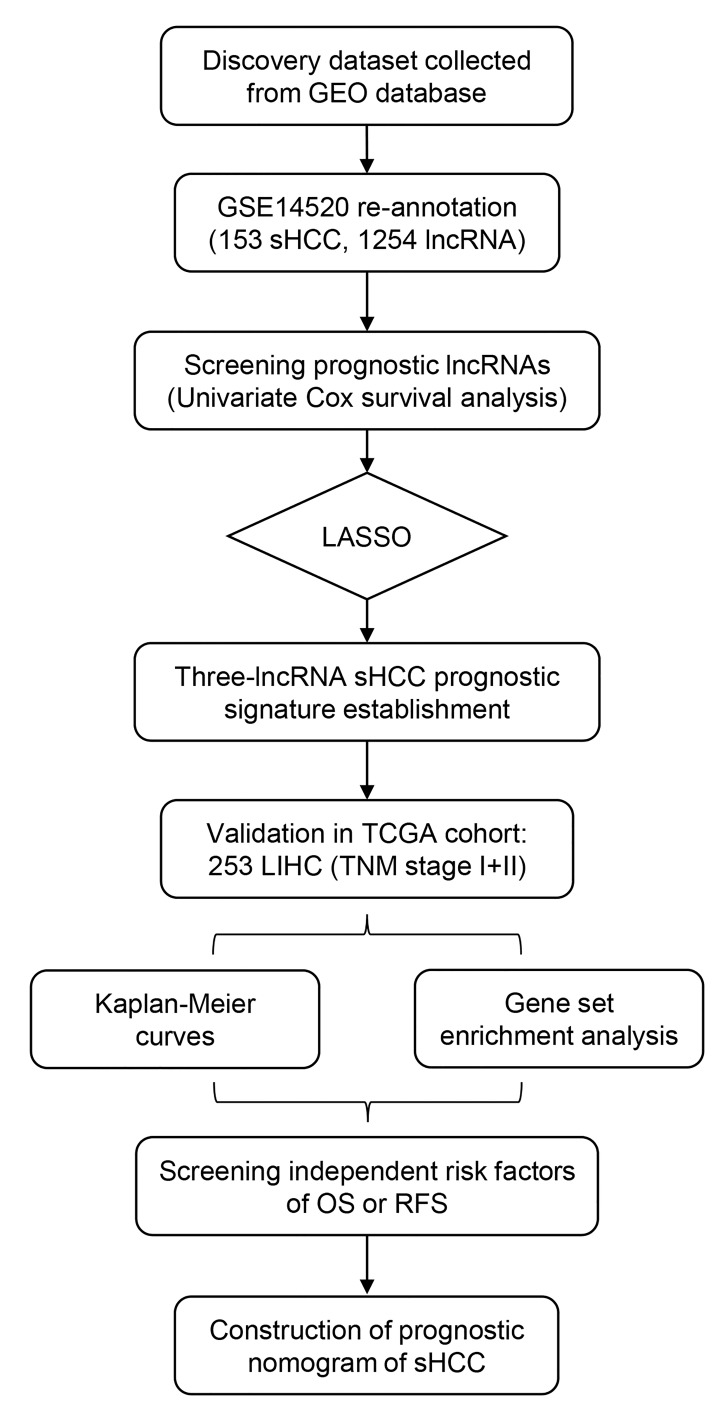
Overview of the analytic pipeline of this study.

**Figure 2:**
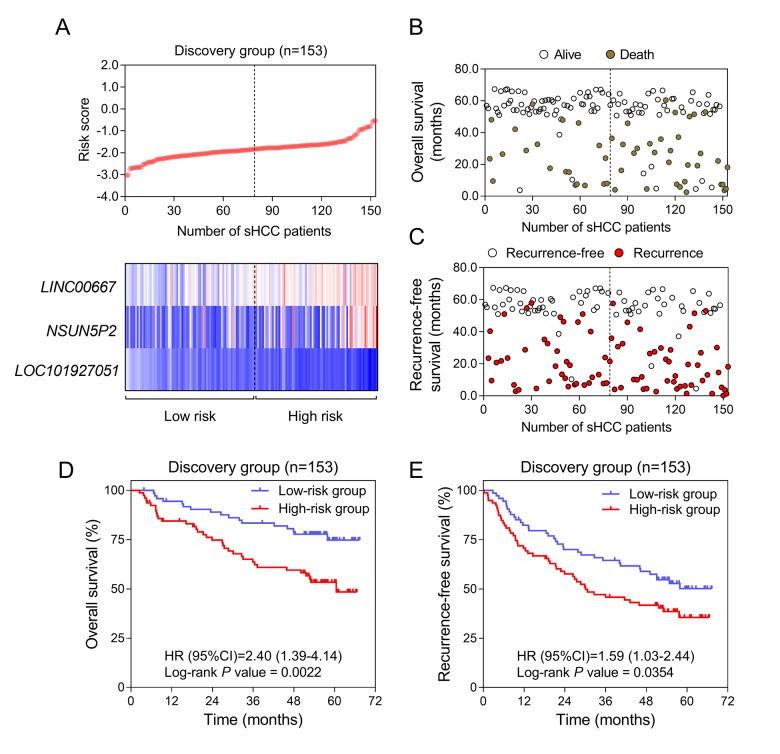
**Construction of the three-lncRNA risk model of sHCC with GSE14250.** (**A**) LncRNA risk score analysis in the discovery series. (*Upper*) LncRNA risk score distribution of 153 sHCC patients. (*Lower*) Expression heatmap of the three lncRNAs corresponding to each sample above. Red: high expression; Blue: low expression. (**B** and **C**) Survival (**B**) and recurrence (**C**) status of every patient in the discovery dataset (N=153). (**D** and **E**) Kaplan-Meier analysis for OS (**D**) and RFS (**E**) using the lncRNA-signature in GSE14520.

The overall design and workflow of this study is presented in Figure 1. After an initial screening of the lncRNAs associated with OS and RFS in the discovery series (GSE14520), the significant lncRNAs (*P* <0.05) were subjected to the LASSO modelling. The sHCC risk score system was built as follows: risk score = (-1.179864) × (expression value of *LOC101927051*) + 0.3570553 × (expression value of *LINC00667*) + 0.1603625 × (expression value of *NSUN5P2*). In this prognostic formula, higher expression of *LOC101927051* was associated with lower risk of death and recurrence (coefficient < 0). On the contrary, higher expression levels of *LINC00667* and *NSUN5P2* were related to worse OS and RFS (coefficient > 0). Based on the absolute value of coefficients, it is not hard to see that *LOC101927051* had the most influence on survival prediction, yet *NSUN5P2* had the least. Using this formula, each patient received a risk score in connection with personal prognosis. Then all patients were classified into high-risk and low-risk groups by the cut-off value of -1.875 based on the risk scores generated from ROC curves (Figure 2A). The OS and RFS in the discovery dataset are presented in Figure 2B and 2C, respectively. The low-risk group was identified to have significantly better clinical outcomes than the high-risk group, in terms of both OS (Log-rank P =0.0022, HR=2.402, 95% CI: 1.392 to 4.143) and RFS (Log-rank P =0.0354, HR=1.588, 95% CI: 1.031-2.444) from KM curves (Figure 2D and 2E).

### Validation and exploration of the risk score model for survival prediction in the TCGA dataset

**Figure 3:**
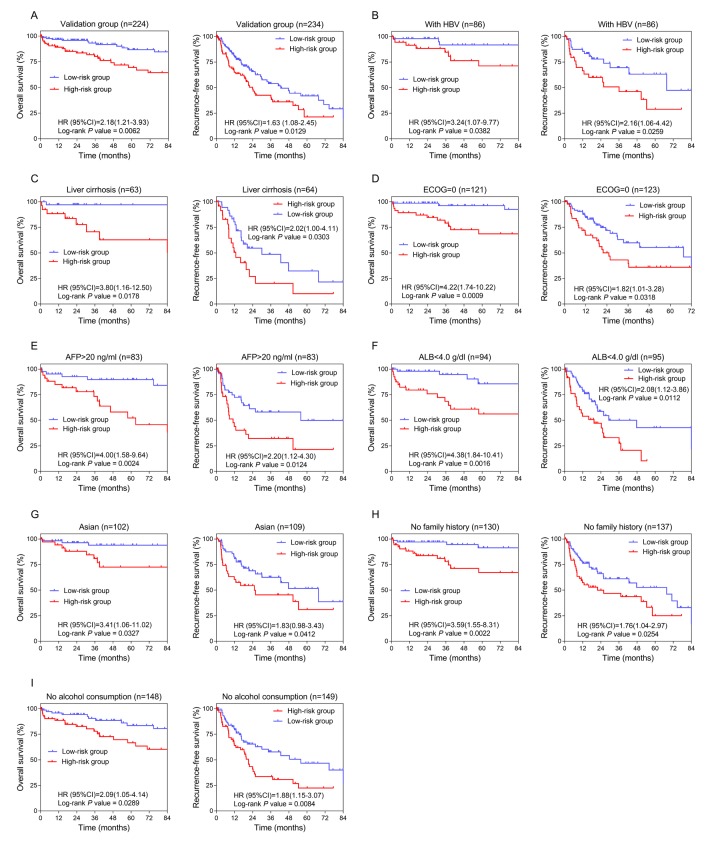
**Confirmation and development of the lncRNA risk score system using the TCGA cohort.** (**A**) Kaplan-Meier analysis for OS (*Left*) and RFS (*Right*) in the validation dataset. (**B**, **C**, **D**, **E**, **F**, **G**, **H** and **I**) Kaplan-Meier analysis for OS (*Left*) and RFS (*Right*) in subgroups stratified by HBV infection (**B**), liver cirrhosis (**C**), ECOG (=0) (**D**), AFP (>20 ng/ml) (**E**), ALB (<4.0 g/dl) (**F**), Asian (**G**), family history (no) (**H**), alcohol consumption (no) (**I)**.

To validate the prognostic value, we applied the three-lncRNA signature to the TCGA cohort (stage I and II). The cut-off points of risk score to divide high-risk and low-risk groups in the validation dataset was 1.33 based on ROC curves. KM curves of the validation series showed great utility in predicting OS and RFS with *P* values from Log-rank tests of 0.0062 (HR=2.183, 95% CI: 1.212-3.932) and 0.0129 (HR=1.627, 95% CI: 1.081-2.451), respectively (Figure 3A).

Analysis was done in TCGA cohort to further investigate the potentiality of the three-lncRNA risk score model. The cut-off value adopted was 1.33, consistent with the overall group. The number of patients classified into high-risk and low-risk groups and the results of Log-rank tests are listed in Table 1. The lncRNA prognostic signature exhibited better performance in HBV and cirrhotic patients with relatively better physical conditions (ECOG =0) (Figure 3B, 3C and 3D). Considering preoperative laboratory indexes, patients with higher serum levels of AFP (alpha-fetoprotein, >20ng/ml) and relatively lower levels of ALB (albumin, <4.0 g/dl) could benefit more in prognosis by using this risk score system (Figure 3E and 3F). As for medical background information, the lncRNA prognostic signature seemed more applicable to Asian patients with no family history of HCC or history of alcohol consumption (Figure 3G, 3H and 3I).

**Table 1:** Stratified analysis of overall and recurrence-free survival in the TCGA samples.

**Characteristics**	**Overall survival**					**Recurrence-free survival**		
	**High-risk / low-risk**		**HR (95% CI)**	***P***		**High-risk / low-risk**	**HR (95% CI)**	***P***
Overall	90/134	2.183 (1.212-3.932)		0.0062*		94/140	1.627 (1.081-2.451)	0.0129*
TNM stage								
Stage I	63/91	2.860 (1.436-5.697)		0.0021*		64/94	1.474 (0.871-2.494)	0.126
Stage II	27/43	1.131 (0.363-3.518)		0.825		30/46	1.922 (1.000-3.693)	0.0329*
Hepatitis								
With HBV	37/49	3.235 (1.071-9.768)		0.0382*		37/49	2.162 (1.056-4.425)	0.0259*
Without HBV	50/74	1.885 (0.922-3.855)		0.065		54/79	1.480 (0.873-2.511)	0.122
Alcohol consumption								
Yes	20/42	1.270 (0.389-4.144)		0.649		23/47	1.170 (0.507-2.703)	0.696
No	67/81	2.086 (1.052-4.136)		0.0289*		68/81	1.876 (1.146-3.071)	0.0084*
Gender								
Male	59/98	1.765 (0.829-3.886)		0.116		62/103	1.670 (0.998-2.794)	0.0317*
Female	31/36	2.627 (1.064-6.483)		0.0349*		32/37	1.485 (0.748-2.951)	0.254
Age								
≤ 60	36/72	1.692 (0.582-4.922)		0.301		39/75	2.039 (1.124-3.699)	0.0081*
> 60	54/62	2.170 (1.079-4.366)		0.0269*		55/65	1.317 (0.744-2.332)	0.331
Liver cirrhosis								
Yes	27/36	3.801 (1.156-12.500)		0.0178*		27/37	2.025 (0.997-4.112)	0.0303*
No	31/52	1.167 (0.444-3.067)		0.744		32/53	1.637 (0.817-3.283)	0.130
Albumin (g/dl)								
< 4.0	40/54	4.378 (1.842-10.410)		0.0016*		40/55	2.081 (1.120-3.865)	0.0112*
≥ 4.0	43/70	1.213 (0.491-3.000)		0.660		44/70	1.161 (0.646-2.085)	0.609
Creatinine (mg/dl)								
< 1.1	55/87	1.854 (0.880-3.907)		0.0771		55/88	1.745 (1.034-2.944)	0.0228*
≥ 1.1	29/39	2.671 (0.897-7.955)		0.0885		30/39	1.381 (0.658-2.900)	0.372
Alpha-fetoprotein (ng/ml)								
≤ 20	44/67	1.562 (0.627-3.891)		0.323		45/68	1.293 (0.725-2.304)	0.355
> 20	35/48	3.898 (1.576-9.641)		0.0024*		35/48	2.196 (1.120-4.304)	0.0124*
Platelet (×10^9^/L)								
< 200	38/69	2.938 (1.167-7.395)		0.0094*		39/70	1.249 (0.670-2.329)	0.466
≥ 200	47/57	1.504 (0.672-3.368)		0.313		47/57	1.712 (0.958-3.059)	0.0555
Race								
Asian	38/64	3.412 (1.057-11.020)		0.0327*		42/67	1.830 (0.978-3.426)	0.0412*
White	42/62	2.153 (1.011-4.589)		0.0324*		42/65	1.501 (0.852-2.646)	0.130
Body mass index								
< 25	45/64	2.928 (1.199-7.151)		0.0107*		48/66	1.399 (0.773-2.532)	0.248
≥ 25	39/65	1.321 (0.553-3.154)		0.521		40/65	1.838 (1.019-3.315)	0.0287*
Family history								
Yes	30/36	1.403 (0.601-3.276)		0.423		30/36	1.432 (0.675-3.041)	0.336
No	56/74	3.594 (1.554-8.311)		0.0022*		60/77	1.755 (1.037-2.970)	0.0254*
ECOG ^a^								
=0	47/74	4.218 (1.741-10.220)		0.0009*		48/75	1.820 (1.010-3.280)	0.0318*
>0	26/42	0.640 (0.242-1.692)		0.371		29/45	1.829 (0.933-3.586)	0.0527
Histological grade								
G1/2	48/89	1.520 (0.677-3.412)		0.278		52/94	1.885 (1.095-3.248)	0.0117*
G3/4	41/44	2.340 (0.992-5.520)		0.0573		41/45	1.284 (0.684-2.408)	0.422
Adjacent tissue inflammation								
Yes	36/46	1.408 (0.497-3.986)		0.506		36/46	1.756 (0.933-3.306)	0.0668
No	27/51	2.067 (0.676-6.321)		0.156		28/51	1.485 (0.726-3.041)	0.240

Abbreviations: HR, hazard ratio; 95% CI, 95% confidence interval. * Statistically significant; ^a^ Eastern Cooperative Oncology Group.

### Identification of relevant biological processes and pathways of the three-lncRNA signature

**Figure 4:**
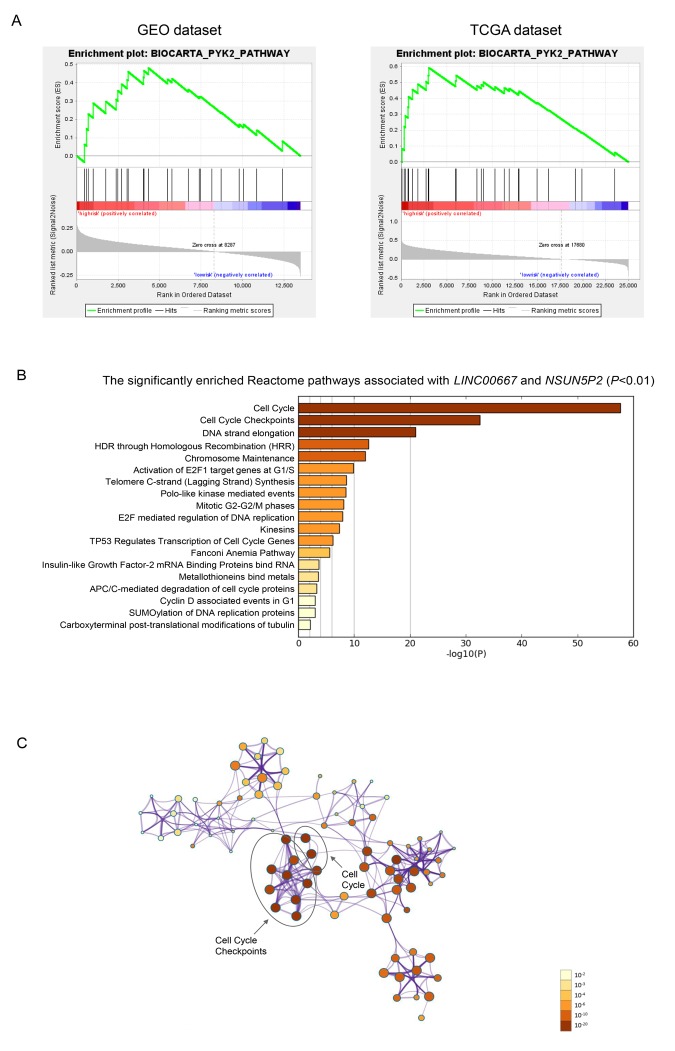
**Gene enrichment analysis of the lncRNA-signature.** (**A**) Gene set enrichment analysis in high-risk patients. Pathway PYK2 was significantly enriched in GEO (A) and TCGA (B) database simultaneously. (**B**) The bar chart of the significantly enriched pathways of the co-expressed genes of *LINC00667* and *NSUN5P2* (P<0.01). (**C**) Correction network of the significant pathway clusters (listed in panel **B**) visualized in Cytoscape. Each cluster was made up of the the best enriched Reactome pathways within the threshold of Kappa-statistical similarity (0.3). Each node represented one enriched term and was colored by P value. In the figure, the top 2 enriched pathways and the clusters they belonged to were marked.

BioCarta pathway enrichment through GSEA was conducted in high-risk groups of both discovery and validation datasets simultaneously. Only PYK2 pathway was significantly enriched in both datasets (Figure 4A). This pathway was reported to play a role in tumorigenesis and tumor progression that might be partly responsible for the poor prognosis of sHCC [24,25]. The two lncRNAs, *LINC00667* and *NSUN5P2*, which indicated a poor prognosis of sHCC, were enriched in the same module. The significantly enriched pathways of *LINC00667* and *NSUN5P2* were mainly associated with cell cycle (Figure 4B and 4C).

### Establishment of nomogram for recurrence-free survival prediction in sHCC

**Figure 5:**
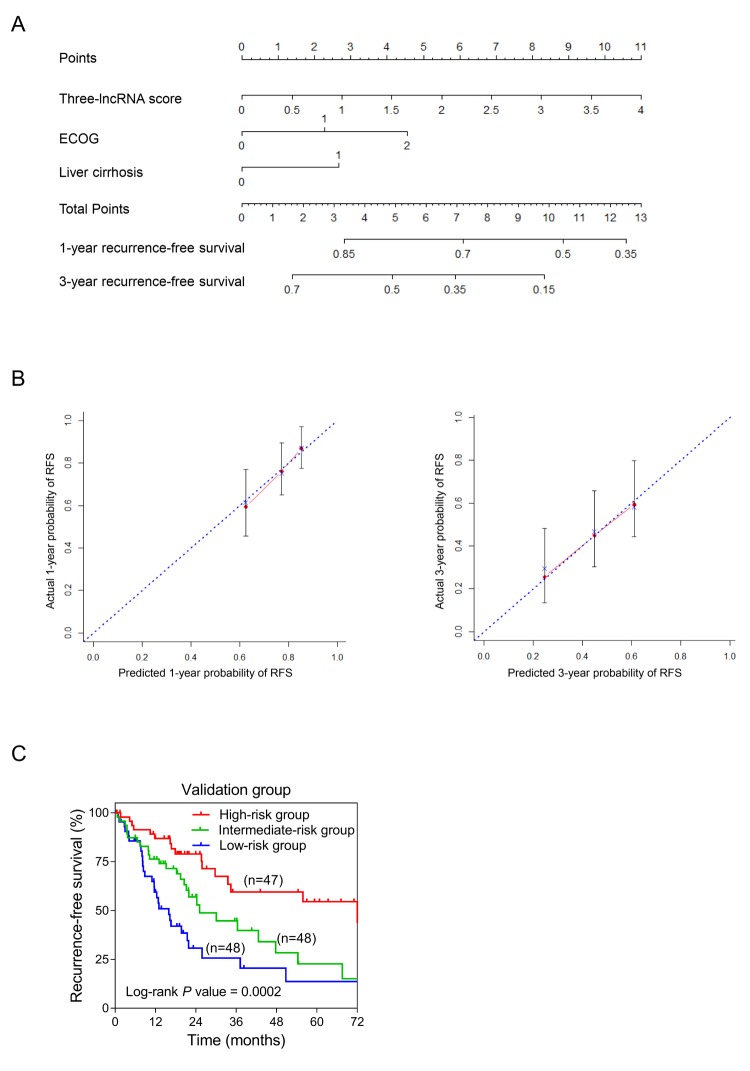
**Establishment of the RFS nomogram for sHCC patients using the TCGA dataset.** (**A**) Nomogram for predicting RFS of sHCC. There are three components in this nomogram: the three-lncRNA score, ECOG and liver cirrhosis. Each of them generates points according to the line drawn upward. And the total points of the three components of an individual patient lie on “Total Points” axis which corresponds to the probability of 1-year and 3-year RFS rate plotted on the two axes below. (**B**) Calibration plots of the nomogram for predicting RFS rate at 1 year (*Left*) and 3 years (*Right*). The predicted and the actual probabilities of RFS were plotted on the x- and y-axis, respectively. (**C**) Kaplan-Meier curves of three risk subgroups stratified by the total points the nomogram gives.

In order to integrate all independent risk factors of OS and RFS for the construction of sHCC prognostic nomogram, various clinicopathological factors including *TP53* mutation and the expression level of *PTK2B*, the core gene of PYK2 pathway, of each TCGA sample were subjected to univariate and multivariate COX regression analyses. The risk score was the significant independent factor of RFS (*P* = 0.004, HR = 1.811, 95% CI: 1.329-2.466) rather than OS (data not shown). Liver cirrhosis and ECOG were also independent risk factors of RFS of sHCC (Table 2). Ultimately, RFS nomogram was formulated based on the three significantly independent factors above. Furthermore, one- and three-year predicted RFS rate was shown in the nomogram (Figure 5A). The C-index for recurrence-free survival prediction was 0.633 (95% CI, 0.562-0.704). The calibration curves of the nomogram showed good agreement between the predicted 1- and 3-year RFS rate and the actual observation (Figure 5B). Each patient with complete clinical information on liver cirrhosis (or not) and ECOG score would get the Nomo-score based on which, patients were classified into different risk groups by the cut-off values 4.35 and 6.25. From Kaplan-Meier analysis of the TCGA dataset, notable differences were observed between high-, intermediate- and low-risk groups (*P* = 0.0002) (Figure 5C).

**Table 2:** Univariate/multivariate COX regression analyses of clinicopathologic factors associated with RFS in the TCGA cohort.

**Variables**	**Univariate analysis**			**Multivariate analysis**	
	**HR (95% CI)**	***P***		**HR (95% CI)**	***P***
Three-lncRNA risk score	1.34 (1.02-1.76)	0.034*		1.74 (1.20-2.52)	0.004*
TNM stage (II/I)	1.76 (1.18-2.62)	0.006*		1.44 (0.89-2.34)	0.14
Gender (male/female)	0.92 (0.60-1.39)	0.68		—	—
Age (>60/≤60 years)	0.87 (0.59-1.29)	0.48		—	—
HBV (Yes/No)	0.67 (0.43-1.02)	0.062		—	—
Alcohol consumption (Yes/No)	0.79 (0.50-1.25)	0.31		—	—
Liver cirrhosis (Yes/No)	1.69 (1.06-2.70)	0.028*		1.71 (1.06-2.75)	0.028*
Albumin (≥4.0/<4.0 g/dl)	1.39 (0.92-2.08)	0.12		—	—
Creatinine (≥1.1/<1.1 mg/dl)	0.70 (0.45-1.09)	0.11		—	—
AFP ^a^ (>20/≤20 ng/ml)	1.18 (0.77-1.80)	0.45		—	—
Platelet (≥200/<200×10^9^/L)	1.16 (0.77-1.74)	0.48		—	—
Race (Asian/not Asian)	0.79 (0.53-1.18)	0.25		—	—
BMI ^b^ (≥25/<25 kg/m^2^)	1.01 (0.68-1.52)	0.95		—	—
Family history (Yes/No)	0.98 (0.62-1.53)	0.92		—	—
ECOG ^c^ (>0/0)	1.49 (1.09-2.04)	0.013*		1.56 (1.03-2.37)	0.036*
Histological grade (G3-4/G1-2)	1.06 (0.71-1.59)	0.78		—	—
Adjacent tissue inflammation (Yes/No)	1.43 (0.90-2.25)	0.13		—	—
*TP53* mutation (Yes/No)	1.21 (0.79-1.85)	0.38		—	—
*PYK2B* expression	1.02 (0.89-1.18)	0.74		—	—

Abbreviations: RFS, recurrence-free survival; HR, hazard ratio; 95% CI, 95% confidence interval.

*:Statistically significant; ^a^:Alpha-fetoprotein; ^b^:body mass index; ^c^:Eastern Cooperative Oncology Group.

## DISCUSSION

Tumor size is an established independent risk factor for HCC that has been applied to staging system for medical guidance. Early-stage patients (solitary tumor ≤5 cm), if receiving proper and timely personalized treatment and surveillance, can have a satisfactory clinical outcome [26]. Moreover, therapeutic approach selection and monitoring indexes are fatal to the prognosis of early-stage HCC [27]. Therefore, reliable biomarkers and genetic signatures as treatment targets and prognostic predictors are of importance for sHCC. After decades of research on genetic markers of cancer-related events like genes and miRNAs, lncRNAs have attracted much attention recently. To the best of our knowledge, this is the first study that have constructed a lncRNA-expression-based risk model to predict the prognosis of sHCC. First and foremost, we repurposed the whole set of microarray probes of GSE14520 for lncRNAs and employed 153 sHCC samples as the discovery dataset. Of all 1254 re-annotated lncRNAs, three (*LOC101927051*, *LINC00667* and *NSUN5P2*) were selected to construct the risk score system for sHCC prognosis through analysis *in silico*. The three-lncRNA signature was eventually validated and developed in another independent cohort from the TCGA.

Functional annotations in high-risk patients of both discovery and validation datasets revealed that PYK2 pathway was significantly enriched. Proline-rich tyrosine kinase 2 (*PYK2*), known as *PTK2B* (protein tyrosine kinase 2 beta), is one of focal adhesion kinases (FAKs) in the regulation of calcium flux of iron channels and activation of cellular signaling pathway like the Canonical (β-Catenin-dependent) Wnt signaling pathway [24,28]. There is evidence that *PTK2B* was involved in cell proliferation, invasion and migration of a variety of malignancies, and its alteration can result in the poor prognosis of HCC [29–32]. Therefore, to integrate all independent factors of OS or RFS by using the nomogram, the expression level of *PTK2B* was taken into consideration, while no association with survival was found. Enrichment analysis of the co-expressed genes of *LINC00667* and *NSUN5P2* revealed that the two lncRNAs might be related to cell cycle. More interestingly, the specific cell cycle genes regulated by *TP53* were also found to be significantly enriched (Figure 4B). Besides, since *TP53* mutation was an acknowledged high risk factor to the tumorigenesis and progression of HCC, we evaluated the relation between the mutation status of *TP53* and OS or RFS as well [33]. Our COX regression analysis indicated that *TP53* alteration might not be a risk factor for sHCC.

The construction of our risk score system may help identify high-risk and low-risk patients experiencing sHCC without invasive examinations, and provide advice to surgeons to aid modifying therapeutic strategies, for example, transplantation or curative section, as there are various treatment options for sHCC patients. To achieve a better prediction ability of prognosis, both genetic and clinicopathological characteristics were incorporated in the nomogram. Ultimately, the RFS nomogram was comprised of the three-lncRNA signature, liver cirrhosis status and ECOG score, which can be obtained from liver biopsy, facilitating examination and doctors’ assessment. According to the prognostic signature or the nomogram, if can be put into clinical practice in the future, high-risk patients of cancer-related death and recurrence can be recognized before surgery, and recommended a more aggressive strategy with strictly pre- and post-operative adjuvant treatment and surveillance. However, additional studies are needed to confirm the risk model in larger groups of patients, and the molecular functions of the three separate lncRNAs in HCC also requires further exploration.

In conclusion, we identified a novel three-lncRNA-expression-based risk model for predicting the prognosis of sHCC patients. Based on survival stratified analysis, this lncRNA risk score system is more suitable for cirrhotic and HBV-infected patients with good ECOG performance, low level of preoperative albumin and high level of AFP. In addition, the lncRNA-signature can help improve our understanding of the carcinogenesis and development of HCC, as well as the clinical decision-making as potential biomarkers and therapeutic targets for sHCC patients.

## MATERIALS AND METHODS

### Microarray datasets preparation and re-annotation

Microarray datasets including gene expression profiles and associated clinical characteristics analyzed in this study were downloaded from publicly available GEO database (https://www.ncbi.nlm.nih.gov/geo/) and The Cancer Genome Atlas (TCGA, http://cancergenome.nih.gov/). GSE14520 from GEO database was conducted by GPL571 (Affymetrix Human Genome U133A 2.0 Array) and GPL3921 (Affymetrix HT Human Genome U133A Array), including 247 HCC samples, 239 paired non-tumor tissue samples and 2 healthy liver samples. Out of them, 153 tumor samples with survival information acquired from sHCC formed the discovery dataset. The inclusion criteria were as follows: pathologically verified HCC tissue; the largest tumor no more than 5cm in diameter; complete follow-up data including overall survival status and time, recurrence status and date. The exclusion criteria were as follows: non-tumor or healthy tissue; lack of histological examination results or pathological results were cholangiocarcinoma, combined hepatocholangiocarcinoma or metastatic liver cancer; the size of tumor larger than 5cm in diameter; incomplete follow-up information. Then, we re-annotated array probes from the Affymetrix Human Genome U133A 2.0 Array to obtain lncRNA profiles, mainly according to the methods proposed by Zhang X et al [34]. After mapping probe set IDs to the NetAffx annotation files, we extracted non-coding protein genes and excluded microRNAs, rRNAs and other short RNAs. Eventually, 1254 lncRNA transcripts including duplicates (different probe IDs may be mapped to the same transcript) were re-annotated. Besides, 235 HCC samples in stage I and II with complete survival and recurrence information from the TCGA formed the validation dataset. Of the 235 patients, 220 had surgery, 1 received liver transplantation, 13 underwent other treatment (no specific information) and 1 was lack of therapeutic records. The TCGA HCC genome profiles contained more than 14,400 lncRNA transcripts and 22,700 mRNA transcripts. All the genomic expression data from the two datasets in this study were from tumor tissue. In addition, the mutation information of gene *TP53* of associated TCGA samples was downloaded as well. The median OS and RFS of the discovery and validation sets were 53.0, 38.7 months and 34.1, 15.9 months, respectively.

### Construction and confirmation of the sHCC-lncRNA risk score

Survival analysis based on univariate COX proportional hazards of each lncRNA annotated in the discovery series was done to screen out those with a significant p value less than 0.1. Then, we used the least absolute shrinkage and selection operator (LASSO) [35] to construct the risk score system based on above selected prognostic lncRNAs. LASSO statistical modelling was performed with “glmnet” package in the R software (version 3.4.0, https://www.r-project.org/), and meanwhile the coefficients of eligible lncRNAs in risk score model were generated based on expression data for each sHCC sample [36]. Absolute value of each coefficient denoted the contribution of corresponding lncRNAs to the prognostic risk score.

The corresponding risk scores for the samples from both discovery and validation datasets were calculated using the risk score system. Patients were divided into high-risk and low-risk groups in either cohort with cut-off values determined by the receiver operating characteristic (ROC) curves (time-independent). The whole group was divided into two subgroups according to the outcome event of each patient (dead or alive). Then ROC curves were plotted based on the risk scores and the survival status of each sample. Risk score was selected as the cut-off value when the area under the curve (AUC) reached its maximum. Kaplan-Meier (KM) curves were plotted, and *P* values and hazard ratio (HR) along with 95% confidence interval (CI) from Log-rank tests and COX regression analyses were calculated to compare survival and recurrence risk between high-risk and low-risk groups. Stratified analysis was conducted to evaluate suitable patients of the sHCC prognostic model in the TCGA cohort. In each sub-group stratified by various clinical characteristics, KM curves were plotted accordingly in the overall group. All ROC and KM curves were plotted by the GraphPad Prism version 7.0 and *P* value less than 0.05 was considered statistically significant.

### Gene set enrichment analysis and functional enrichment analysis

Functional annotations in both high-risk and low-risk samples were done through gene set enrichment analysis (GSEA), an approach *in silico* performed by the JAVA program (http://www.broadinstitute.org/gsea) using Molecular Signature Database (MSigDB) [37]. Pathway enrichment was carried out in the high-risk patient group based on the BioCarta pathway database [38]. The significance threshold of false discovery rate (FDR) for the significantly enriched biological processes and pathways was set at 0.05. Gene enrichment analysis of the identified lncRNAs was carried out in the Reactome pathway database using Metascape, a free online tool for gene annotation (http://metascape.org/gp/index.html#/main/step1) [39]. The correction network of the enriched terms was presented in Cytoscape [40]. The possible functional Reactome pathways were enriched based on the co-expressed genes of the lncRNAs in the same module clustered by Weighted Gene Coexpression Network Analysis (WGCNA). WGCNA was a new method for detecting the highly connected genes and conducted with “wgcna” package in R studio [41].

### Statistical analyses

Statistical analyses were conducted with STATA software version 12.0 (StataCorp, TX, USA), unless otherwise indicated. *P* value less than 0.05 was considered statistically different. Univariate and multivariable COX proportional hazards regression analyses were performed in TCGA cohort using risk score and clinical information to find the independent predictor of the OS and the RFS of sHCC. *P*-value less than 0.05 was adopted as a threshold. The nomogram was built based on the significant factors by the package of “rms” in R studio. The concordance index (C-index) and the calibration curves were utilized to evaluate the performance of the nomogram and compare the predicted- and actual- probability of survival. Each patient got the total points from the nomogram (Nomo-score). KM curve analysis was carried out to measure the performance of the nomogram by dividing patients into high-, intermediate- and low-risk groups using tertiles of the Nomo-scores as the cut-off values.
